# Phonon spectrum and thermoelectric properties of square/octagon structure of bismuth monolayer

**DOI:** 10.1039/d0ra08838b

**Published:** 2021-01-27

**Authors:** C. Y. Wu, X. L. Li, J. C. Han, H. R. Gong, S. F. Zhou

**Affiliations:** Department of Educational Science, Hunan First Normal University Changsha Hunan 410205 China; State Key Laboratory of Powder Metallurgy, Central South University Changsha Hunan 410083 China gonghr@csu.edu.cn; Institute of Advanced Wear & Corrosion Resistant and Functional Materials, Jinan University Guangzhou 510632 China; Department of Mechanical Engineering and Materials Science, University of Pittsburgh Pittsburgh Pennsylvania 15213 USA

## Abstract

First-principles calculation and Boltzmann transport theory have been combined to comparatively investigate the band structure, phonon spectrum, lattice thermal conductivity, electronic transport property, Seebeck coefficient, and figure of merit of square/octagon (s/o)-bismuth monolayer. Calculations reveal that the thermoelectric properties of s/o-bismuth monolayer are better than that of β-bismuth monolayer, which should be mainly due to the low lattice thermal conductivity and weakened coupling of electrons and phonons. It is also found that the phonon frequency and group velocity could play dominant roles in determining the magnitude of the lattice thermal conductivity of s/o-bismuth monolayer. Furthermore, the Seebeck coefficient and figure of merit of s/o-bismuth monolayer are higher than those of β-bismuth monolayer. The derived results are in good agreement with other theoretical results in the literature, and could provide a deep understanding of thermoelectric properties of the bismuth monolayer materials.

## Abstract 2

First-principles calculation and Boltzmann transport theory have been combined to comparatively investigate the electronic structure, phonon spectrum, and thermoelectric properties of square/octagon (s/o)-bismuth monolayer.

## Graphical abstract

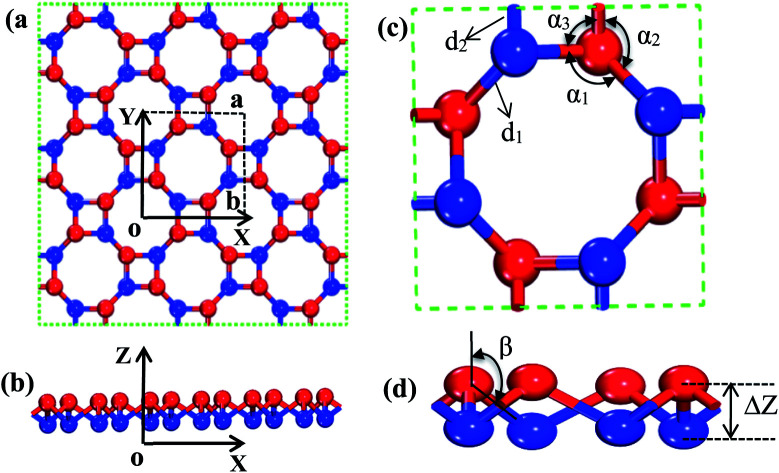

## 1. Introduction

Thermoelectric materials have attracted extraordinary scientific and technological interests during the past decades owing to their unique transport properties and potential applications for power generators, cooling devices, and sensors.^1,2^ Specifically, the unusual electronic structure of the weak overlap and high mobility owing to the highly anisotropic electron ellipsoids of bismuth (Bi), resulting from its highly anisotropic electron ellipsoids, makes it one of the most potential thermoelectric materials.^3^ The properties of thermoelectric materials can be quantified using the dimensionless thermoelectric figure of merit according to the following formula^4^1
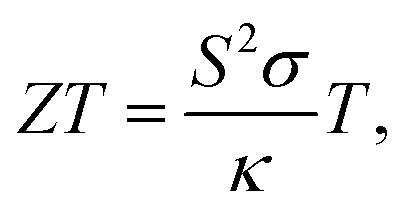
where *S*, *σ*, *T*, and *κ* are the Seebeck coefficient, electrical conductivity, absolute temperature, and total thermal conductance, respectively. Therefore, research on bismuth is mainly focused on regulating the band structure to improve the *ZT* value for high conversion efficiency by achieving high *S* and *σ* values and low thermal conductivity during the past several years.^5–7^ Unfortunately, the coupling of the above transport coefficients makes it challenging to significantly improve the overall thermoelectric performance.

Recently, low dimensionality, as one of the most effective strategies, has been proposed to achieve high thermoelectric performance by tuning band structure and decreasing the lattice thermal conductivity due to the quantum size effects.^8^ Thus, there is an explosive growth of interest for the exploration of two-dimensional (2D) materials of bismuth monolayers, *i.e.*, α (rectangular of interunit Bi–Bi bond), β (hexagon of interunit Bi–Bi bond), γ-bismuth (rectangular of interunit Bi–Bi bond), δ-bismuth (rectangular of interunit Bi–Bi bond), ε (square of interunit Bi–Bi bond), s/o (square/octagon Bi ring), ξ (square of interunit Bi–Bi bond), η (pentagon of interunit Bi–Bi bond), θ (pentagon of interunit Bi–Bi bond), and ι (hexagon of interunit Bi–Bi bond),^9–12^ which potentially have suitable electronic and phonon structures and good thermoelectric properties.^13^ In particular, s/o-bismuth monolayer, which consists of a buckled square and octagon ring lattice, has a suitable band gap (0.34 eV) as well as is thermally stable for applications in the fields of thermoelectric components and electronic devices at room temperature and above.^14^

As acknowledged, the electronic structure and phonon spectrum are essential to determine the figure of merit of thermoelectric materials.^2^ In this respect, the investigation of the s/o-bismuth monolayer in the literature is mainly focused on structural parameters and electronic structures in recent years, while these results from various groups are not consistent with each other.^14–17^ For instance, the bandgap of the s/o-bismuth monolayer covers a wide range from 0.15 to 1.01 eV according to several theoretical studies.^14,15,17^ However, in terms of the thermoelectric properties of the s/o-bismuth monolayer and the underlying reason for the different thermoelectric properties of s/o-bismuth monolayer from its special band structure and phonon spectrum, further research should be conducted on these problems.

In this work, the electronic structure and thermoelectric properties of the s/o-bismuth monolayer are investigated by combining the first-principles calculation and Boltzmann transport theory. The corresponding properties of the β-bismuth monolayer in the literature^18^ are also derived for the sake of comparison by the same calculated method. Specifically, the band structure, phonon spectrum, lattice thermal conductivity, relaxation time, electronic transport properties, Seebeck coefficients, and figure of merits of s/o-bismuth monolayer are calculated. The fundamental impacts of electronic structure and phonon spectrum on the thermoelectric properties of the s/o-bismuth monolayers are revealed and discussed extensively to provide a deep understanding of various properties of the bismuth monolayer.

## 2. Theoretical methods

The optimized atomic structure, phonon spectrum, and electronic structure of the s/o-bismuth monolayer are calculated by means of the well-established Vienna *ab initio* simulation package (VASP) within the density functional theory (DFT).^19,20^ The calculations are performed in a plane-wave basis with the projector-augmented wave (PAW) method.^21–23^ The local density approximation (LDA) with the inclusion of spin–orbit coupling (SOC) is chosen for the exchange and correlation functions,^24,25^ which was proved to be effective in electronic structure calculation of the A7 structure (rhombohedral, space group no. 166. *R*3̄*m*) of Bi and bismuth monolayer in the literature.^3,26,27^

**Fig. 1:**
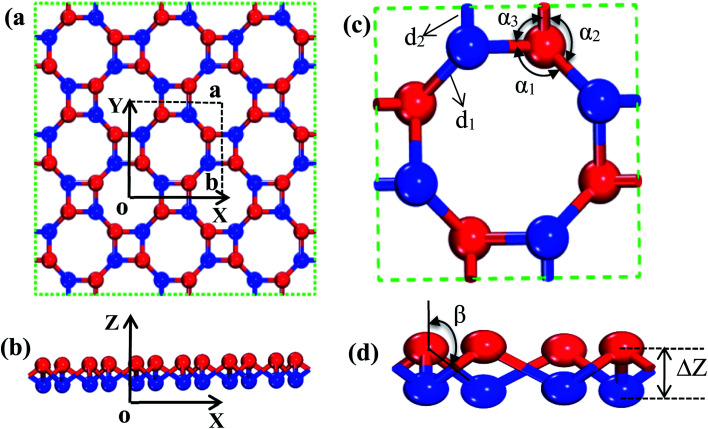
Description of the atomic configuration and structural parameters of buckled s/o-monolayer. (a) Top-views of the square and octagon rings with the lattice constants *a* = *b* as shown with dashed lines. (b) Side-view of the s/o-monolayer with the two parallel atomic planes. (c) An octagon ring of the s/o structure with relevant bonds *d*_1_ and *d*_2_ and bond angles *α*_1_, *α*_2_, and *α*_3_. (d) Side view of the octagon showing the buckling parameter Δ*Z* and bond angle *β*.

The s/o-bismuth monolayer with the buckled square and octagon ring structure containing eight bismuth atoms shown in Fig. 1 is used for the calculations of the lattice structure, band structure, phonon spectrum, and lattice thermal conductivity. After a series of test calculations, the vacuum distance of the s/o-bismuth monolayer is set as 15 Å to avoid the interactions between the layer and its periodic images. In addition, the k-meshes of 11 × 11 × 1, 29 × 29 × 1, and 3 × 3 × 1 are selected for the calculations of the lattice relaxation, the electronic structure and transport properties, and the phonon spectrum of the s/o-bismuth monolayer, respectively. In addition, the energy criteria are 0.001 and 0.01 meV for electronic and ionic relaxations, respectively.

The transport properties of the s/o-bismuth monolayer are derived by means of the Boltzmann transport theory and the rigid band approach (RBA) as included in the software of Boltztrap.^28^ The energy eigenvalues are employed on a very dense nonshifted 12615 *k*-point mesh in the full Brillouin zone (BZ) from the self-consistent converged electronic structure calculations. The transport properties are derived as a function of temperature and chemical potential employing the constant relaxation time approximation (CRTA), which neglects the weak energy dependence of relaxation time (*τ*) but retains some temperature and doping dependence.^29,30^ The effects of temperature and carrier density (*n*) are simulated using the rigid band approximation,^31–33^ which assumes that the effects do not change the shape of the band structure, but only shift the Fermi energy.^18,34^

The lattice thermal conductivity and phonon spectrum of the s/o-bismuth monolayer are calculated by using the Boltzmann transport equations for the phonons as implemented in ShengBTE code^35^ and PHONOPY package.^36^ To obtain the phonon spectrum and lattice thermal conductivity, the second-order harmonic interatomic force constants (IFCs) are calculated using the density-functional perturbation theory (DFPT) with a 4 × 4 × 1 supercell.^37^ In addition, the third-order anharmonic IFCs are performed by using a 4 × 4 × 1 supercell and the interactions up to the fifth nearest neighbors are taken into account with the finite difference method for calculating the lattice thermal conductivity,^38^ while the k-meshes of 3 × 3 × 1 are selected for the supercell calculation with the ring lattice of square and octagon.

## 3. Results and discussion

### 3.1 Band structure of s/o-bismuth monolayer

In order to find out the band structure of the s/o-bismuth monolayer, the structural parameters (*a*, *d*_1_, *d*_2_, *α*_1_, *α*_2_, *α*_3_, *β*, and Δ*Z*) of the s/o-bismuth monolayer are first optimized using the PAW + LDA + SOC method, which can describe the band structure of Bi more accurately than the generalized gradient approximation with the PAW method.^18,21,39^ Accordingly, Fig. 1(a)–(d) show the derived atomic configuration and its parameters of the buckled s/o monolayer from different perspectives. The whole structure consists of two atomic planes of the buckled square and octagon rings and all the corners of the rings are occupied by the atom of bismuth.

The calculated lattice constants (*a*), bond lengths (*d*_1_ and *d*_2_), bond angle (*α*_1_, *α*_2_, *α*_3_, and *β*), and buckling parameter (Δ*Z*) of the s/o-bismuth monolayer are summarized in Table 1. The available theoretical results of the s/o-bismuth monolayer in the literature^14,15,17^ and β-bismuth monolayer are also included in Table 1 for comparison. It can be clearly seen from this table that the optimized lattice parameters for the s/o-bismuth monolayer using the present PAW + LDA + SOC method are in good agreement with the previously reported results using other methods.^14,15,17^ In addition, one can also discern that the bond lengths (*d*_1_ and *d*_2_) and buckling parameter (Δ*Z*) of the s/o-bismuth monolayer is slightly less than that of the corresponding β-bismuth monolayer.^18^

**Table 1:** Optimized lattice constant *a* and *b* (Å), bond length *d*_1_ (Å), buckling parameter Δ*Z* (Å), and unit volume (*V*) of the s/o-bismuth monolayer and β-bismuth monolayer

Structure	*a*	*d*_*i*_	*α*_*i*_, *β*	Δ*Z*	Method	Ref
s/o-Monolayer	8.46	*d*_1_ = 2.996, *d*_2_ = 3.020	*α*_1_ = 98.993, *α*_2_ = 98.993, *α*_3_ = 71.335, *β* = 130.309	1.708	LDA + SOC	This work
s/o-Monolayer	8.40	*d*_1_ = 2.990, *d*_2_ = 3.080	*α*_1_ = 96.60, *α*_2_ = 96.60	1.780	PBE + SOC	15
s/o-Monolayer	8.74	*d*_1_ = 3.044, *d*_2_ = 3.059	*α*_3_ = 70.40 *β* = 126.70	1.757	PBE + SOC	17
s/o-Monolayer	8.74	*d*_1_ = 3.020, *d*_2_ = 3.050		1.760	GGA + SOC	14
β-Monolayer	4.54	*d*_1_ = 3.05		1.730	LDA + SOC	18

**Fig. 2:**
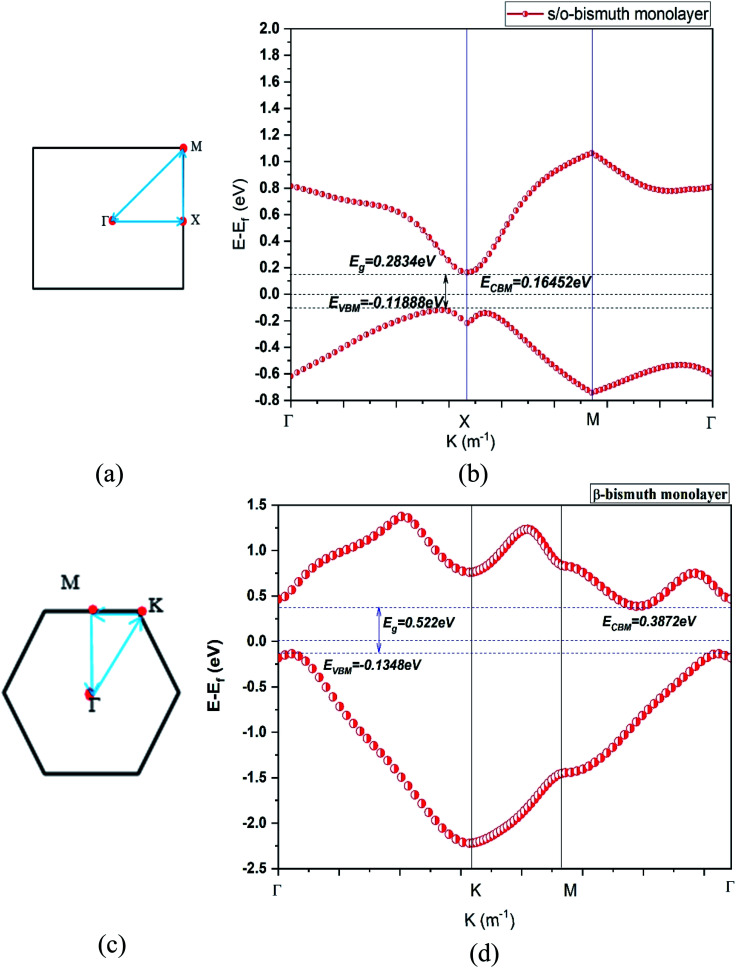
The first Brillouin zone (a) (c) and the band structure (b) (d) of the s/o-bismuth and β-bismuth monolayer^18^ using the LDA + SOC method, respectively.

To figure out the impact of the electronic structure on the electric transport performance, the band structure and band gap of the s/o-bismuth monolayer are calculated and shown in Table 2 and Fig. 2. Additionally, the band structure of the β-bismuth monolayer in our previous work^18^ and the related theoretical band structure of the s/o-bismuth monolayer in the literature^14,15,17^ are also included in Table 2 for the sake of comparison.

**Table 2:** Valence band maximum *E*_VBM_, conduction band minimum *E*_CBM_, and the bandgap *E*_g_ of the s/o-bismuth monolayer and β-bismuth monolayer

Structure	*E*_VBM_ (eV)	*E*_CBM_ (eV)	*E*_g_	Method	Reference
s/o-Monolayer	−0.1189	0.1645	0.283	LDA + SOC	This work
s/o-Monolayer			0.34	PBE + SOC	15
s/o-Monolayer			0.63	PBE	15
s/o-Monolayer			0.15	HSE + SOC	15
s/o-Monolayer			1.01	HSE	15
s/o-Monolayer			0.41	PBE + SOC	17
s/o-Monolayer			0.33	PW91 + SOC	14
β-Monolayer	−0.1348	0.3872	0.522	LDA + SOC	18

First of all, the band gap (0.283 eV) of the s/o-bismuth monolayer using the PAW-LDA-SOC method agrees well with the reported values 0.330 and 0.340 eV or 0.41 eV from the GGA-PW91-SOC and GGA-PBE-SOC methods,^14,15^ respectively, while larger than the value of 0.150 eV from the HSE-SOC method.^17^ Interestingly, the band gap (0.283 eV) of the s/o-bismuth monolayer from the present PAW-LDA-SOC method is less than the calculated values of 0.63 eV and 1.01 eV from other theoretical methods of PBE and HSE, respectively.^15^

Secondly, it can be clearly observed from Fig. 2(a) and (b) that the main shape of the band structure of the s/o-bismuth monolayer, the electrons and holes of Fermi surfaces centered at the *X* points and along the *Γ*–*X* high symmetrical path in the Brillouin zone are in good agreement with the corresponding reported results in the literature.^17^ Note that the *X* points and energy bands along the *Γ*–*X* directions in the s/o-bismuth monolayer are very close to *E*_f_ and thus dominate the main features of its electronic structure and transport properties. However, the energy bands along the *Γ*–*M* and *Γ*–*K* direction in the β-bismuth monolayer are near *E*_f_ and play a decisive role according to Fig. 2(c) and (d).

Thirdly, it can be seen from Fig. 2(b) and (d) that the valence band maximum (VBM) and the conduction band minimum (CBM) of the s/o-bismuth monolayer are lower and higher than those of β-bismuth monolayer, respectively. Additionally, both s/o-bismuth and β-bismuth monolayers are typical indirect semiconductors. Moreover, the bandgap of the s/o-bismuth monolayer is 0.283 eV, which is smaller than the corresponding value of 0.522 eV of the β-bismuth monolayer calculated using the same PAW-LDA-SOC method.

### 3.2 Phonon spectrum and lattice thermal conductivity of s/o-bismuth monolayer

**Fig. 3:**
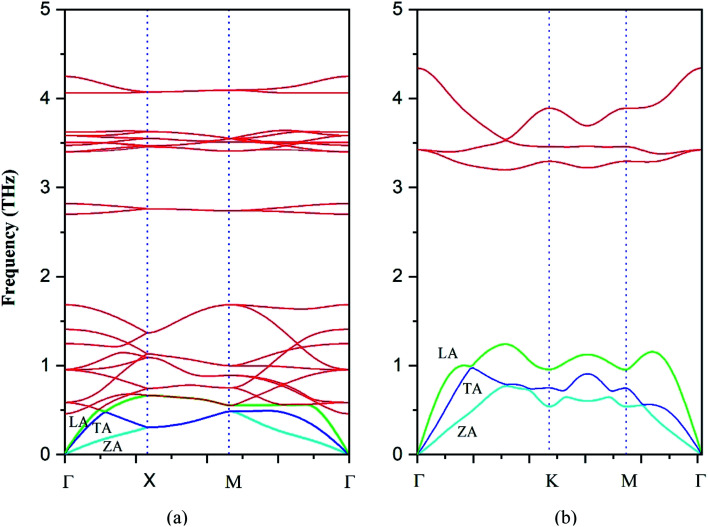
Phonon band structure of the s/o-bismuth monolayer (a) and β-bismuth monolayer^18^ (b).

It is of value to fundamentally understand the phonon transport properties of the s/o-bismuth monolayer. First of all, the phonon spectrum of the s/o-bismuth monolayer is calculated and shown in Fig. 3 by the finite difference method implemented in the Phonopy package.^36^ The corresponding result of the β-bismuth monolayer is also included in Fig. 3 (ref. 18) for the sake of comparison. One can discern from Fig. 3 that the phonon spectrum of the s/o-bismuth monolayer is free from imaginary frequencies in the first Brillouin zone, indicating that this structure is thermodynamically stable, which is in good agreement with the other previous works.^14,15,17^ Moreover, the longitudinal acoustic (LA) and transverse acoustic (TA) branches of the s/o-bismuth monolayer are linear when the wave vector *q* is close to the *Γ* point. However, the acoustic (ZA) branch of the s/o-bismuth monolayers in the *z*-direction deviates from linearity near the *Γ* point owing to the sufficiently weak interplanar interactions by the microscopic elastic theory, which is a generic feature of the monolayer material including the β-bismuth monolayer.^40–42^

**Fig. 4:**
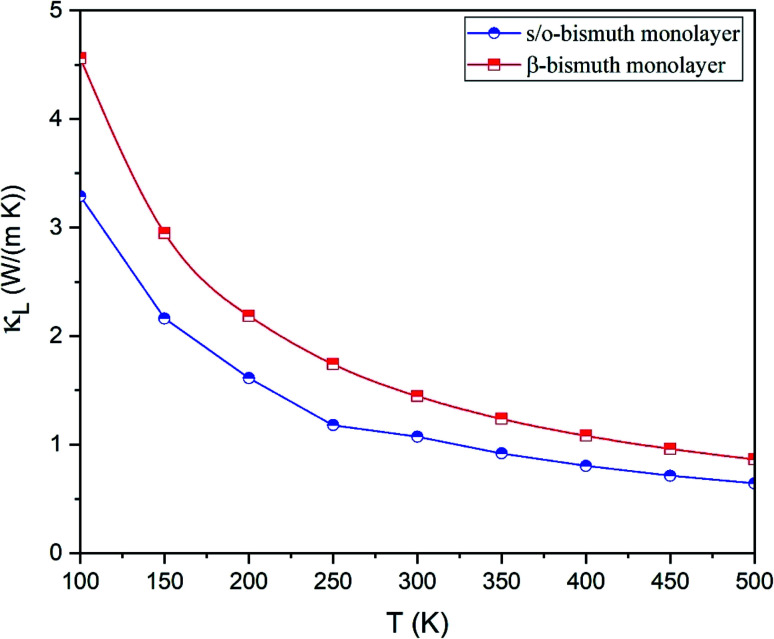
Lattice thermal conductivity of s/o-bismuth monolayer and β-bismuth monolayer^18^ as the function of temperature with respect to a uniform vacuum thickness of 15 Å.

The lattice thermal conductivity (*κ*_l_) of the s/o-bismuth monolayer is then calculated as a function of temperature in the framework of the supercell approach through the obtained second- and third-order interatomic force constants as implemented in the ShengBTE.^35^ Consequently, Fig. 4 shows the derived lattice thermal conductivity (*κ*_l_) of the s/o-bismuth monolayer, including the β-bismuth monolayer as the function of temperature for the sake of comparison. One can observe from this figure that the lattice thermal conductivity of s/o-bismuth is significantly lower than that of the β-bismuth monolayer, which indicates the important role of the structure in determining the lattice thermal conductivity of the bismuth monolayers.

It is of vital importance to have a deep understanding of the intrinsic reason why the lattice thermal conductivity of the s/o-bismuth monolayer is lower than that of the β-bismuth monolayer. Accordingly, the lattice thermal conductivity of the bismuth monolayer may be derived from the summation of the contribution of all the phonon modes from the phonon kinetic theory in the following form:^43^2
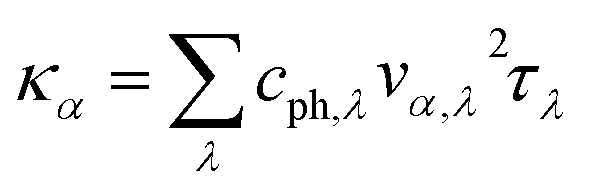
where *κ*_*α*_, *c*_ph,*λ*_, *v*_*α*,*λ*_, and *τ*_*λ*_ are the lattice thermal conductivity in the *α* direction, the phonon volumetric specific heat of each other, the phonon group velocity of mode *λ* along the *α* direction, and the phonon lifetime of the mode *λ*, respectively. Obviously, the lattice thermal conductivities of the s/o-bismuth and β-bismuth monolayers are proportional to *τ*_*λ*_, *ν*_a,*λ*_^2^, and *c*_ph,*λ*_ in eqn (2).

**Fig. 5:**
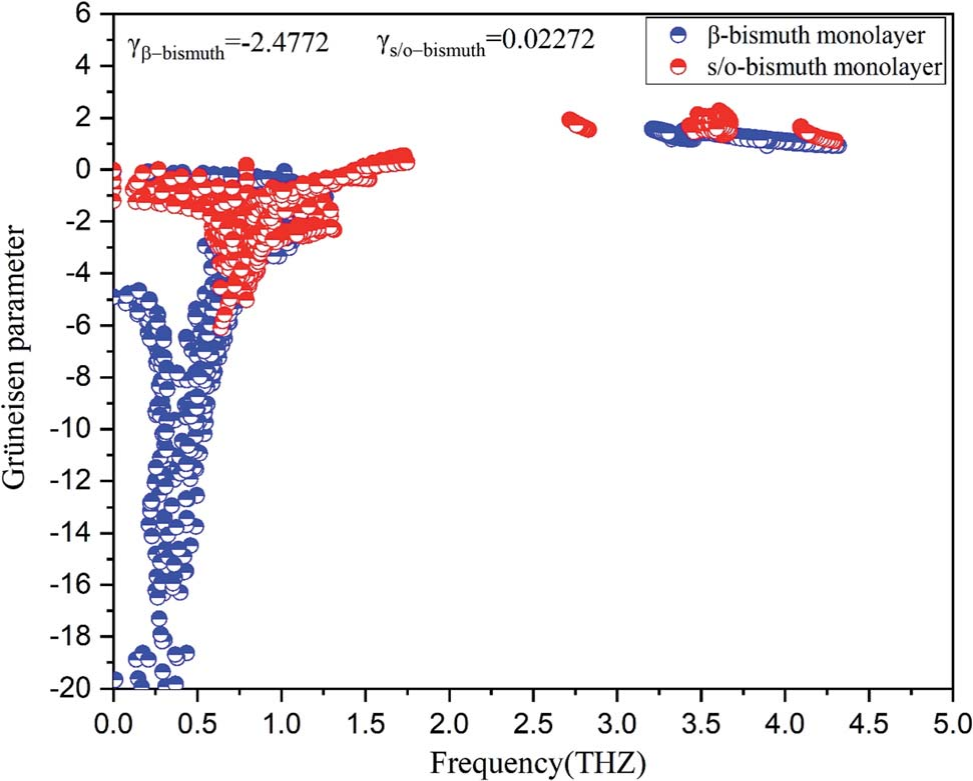
Grüneisen parameters of s/o-bismuth and β-bismuth monolayers^18^ as the function of the frequency at the 300 K.

To investigate the effect of phonon scattering on the lattice thermal conductivity, the three-phonon scattering, including both the strength of each scattering channels and the phonon scattering channels are calculated, which are described by the Grüneisen parameter (*γ*) and the total phase space for three-phonon processes (P_3_), respectively. The Umklapp scattering time, clarified as the *τ*_U_, is inversely proportional to *γ*^2^ as follow showing the contribution of Grüneisen parameter:^44^3
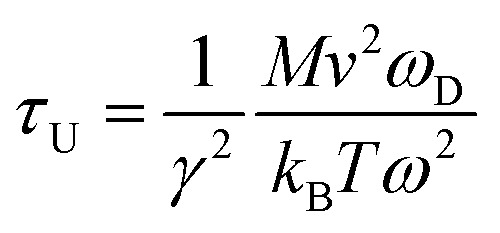
where *M*, *v*, and *ω*_D_ are the mass of the unit cell, the phonon group velocity, and Debye frequency, respectively. It should be noted that the Grüneisen parameter describes the strength of each scattering channel, which depends on the degree of anharmonicity of the phonon mode.^45^ Accordingly, the Grüneisen parameters (*γ*) of the s/o-bismuth and β-bismuth monolayers as a function of frequency at 300 K are shown in Fig. 5, in which the Grüneisen parameters (*γ*) averaged over all phonons are −2.4772 and 0.02272 for the β-bismuth and s/o-bismuth monolayers from the ShengBTE results, respectively. It can be obviously seen from Fig. 5 that the square of the Grüneisen parameter (*γ*^2^) of the β-bismuth monolayer is larger than that of the s/o-bismuth monolayer, especially in the low-frequency region of the acoustic phonons.^46^ In this view, the lattice thermal conductivity of the s/o-bismuth monolayer should be higher than that of the β-bismuth monolayer because its phonon lifetime should be longer with the small *γ*^2^ according to eqn (3), which is inconsistent with the above-obtained results of the lattice thermal conductivity. Therefore, the impact of the Grüneisen parameter on the lattice thermal conductivity of the s/o-bismuth and β-bismuth monolayers can be negligible or can be counteracted by other dominant factors.

**Fig. 6:**
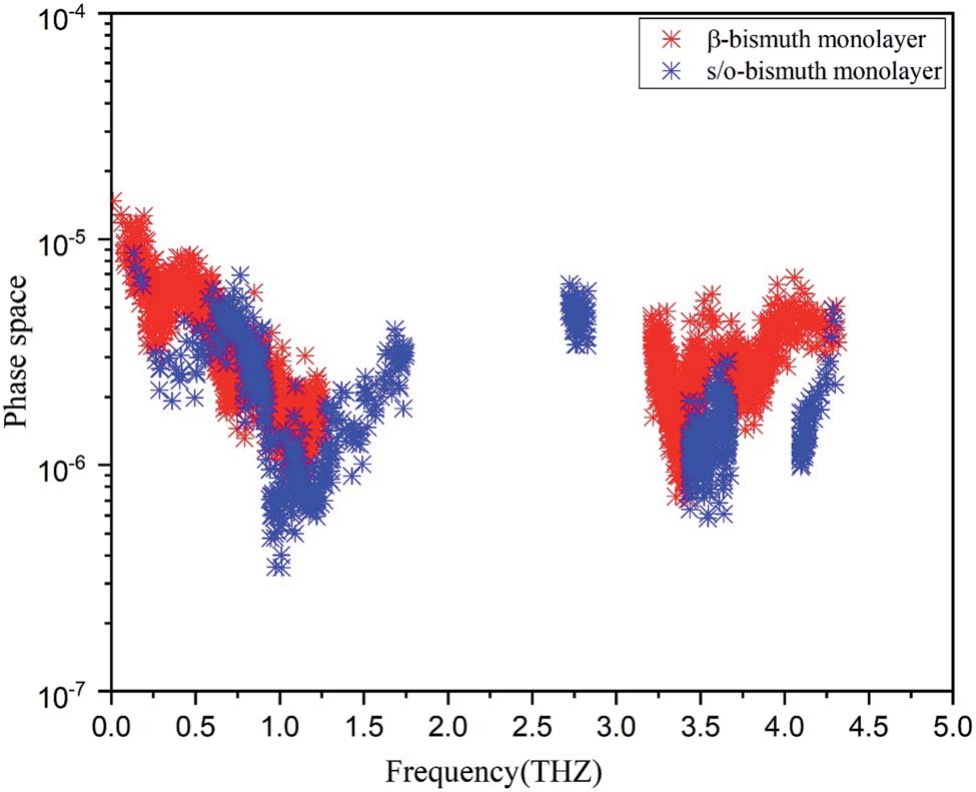
Scattering phase space of all modes in s/o-bismuth and β-bismuth monolayers^18^ as the function of the frequency at 300 K.

In addition, the total phase spaces for three-phonon processes (P_3_) of all modes in s/o-bismuth and β-bismuth are calculated and shown in Fig. 6. Interestingly, the channels of the scattering phase space of the s/o-bismuth monolayer (P_3_) are lower than those of the β-bismuth monolayer. The larger P_3_ typically indicates more channels of phonon scattering, a larger scatter rate, and consequently, lower intrinsic lattice thermal conductivity,^47^ which is contrary to our above-calculated lattice thermal conductivity results. Therefore, it could be deduced that both the strength of each scattering channels and the phonon scattering channels of the three-phonon scattering process cannot explain the unexpected phonon transport behavior in bismuth monolayers.

**Fig. 7:**
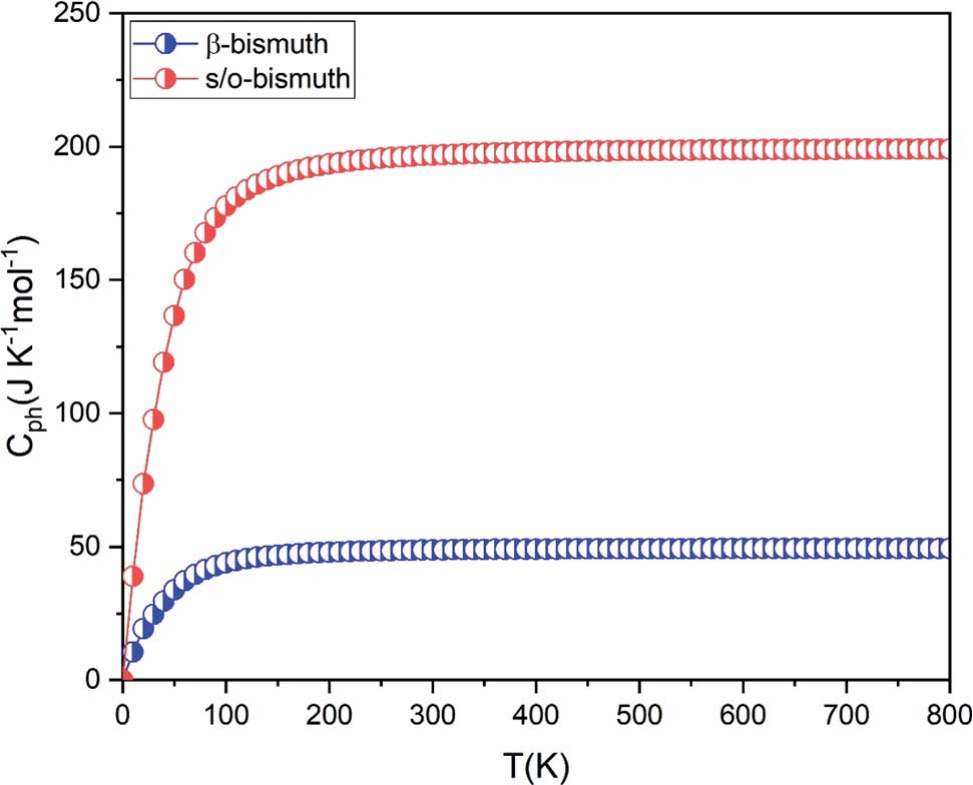
The phonon volumetric specific heat of s/o-bismuth and β-bismuth monolayers^18^ as the function of temperature.

If the differences of three-phonon scattering cannot be attributed, the differences between the phonon volumetric specific heat and the group velocity must be the governing factors of the lattice thermal conductivity according to eqn (2). The phonon volumetric specific heats of the s/o-bismuth and β-bismuth monolayers can be calculated by the following formula:^43^4
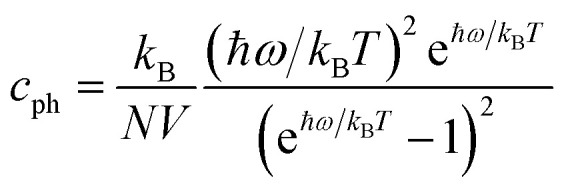
where *k*_B_, ℏ, *ω*, *T*, *N*, and *V* are the Boltzmann constant, the reduced Planck constant, the phonon angular frequency, the absolute temperature, the number of *q* points in the first Brillouin zone, and the volume of the unit cell, respectively. Accordingly, the phonon volumetric specific heats of the s/o-bismuth and β-bismuth monolayers are derived and shown in Fig. 7 for the sake of comparison. It can be clearly seen from Fig. 7 that the phonon volumetric specific heat of the s/o-bismuth monolayer is larger than that of the β-bismuth monolayers, implying that the phonon volumetric specific heat must not be the governing factor of the lower lattice thermal conductivity of s/o-bismuth monolayer according to eqn (2). Thus, the effects of the phonon group velocity on the lattice thermal conductivity become the only remaining crucial factor to understand the intrinsic reason for the different lattice thermal conductivity of the s/o-bismuth and β-bismuth monolayers.

The group velocity could be defined in the following form:^48^5
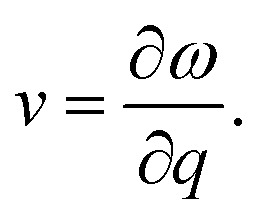


After a series of calculations, Table 3 summarizes the maximum frequency of ZA, TA, and LA from the calculated phonon spectra of the s/o-bismuth monolayer, as shown in Fig. 3 as well as the β-bismuth monolayer for the sake of comparison. Furthermore, the corresponding maximum group velocities of ZA, TA, and LA are also obtained and listed in Table 3 according to eqn (5). It can be clearly observed from Table 3 that the maximum frequencies of ZA, TA, and LA and the maximum group velocity of the s/o-bismuth monolayer are lower than those of the β-bismuth monolayer, suggesting that lower phonon velocity and acoustic-phonon frequency lead to the lower lattice thermal conductivity of the s/o-bismuth monolayer. Overall, the lower lattice thermal conductivity of the s/o-bismuth monolayer than the β-bismuth monolayer is owing to the lower phonon velocity (*v*) and acoustic-phonon frequency (*ω*). On the contrary, the influence of the Grüneisen parameter (*γ*), the total phase space for three-phonon processes (P_3_), and the phonon volumetric specific heat (*c*_ph_) on the lattice thermal conductivity are negligible based on our results.

**Table 3:** The maximum frequency of the longitudinal acoustic branch (*ω*^m^_LA_), the maximum frequency of the transverse acoustic branch (*ω*^m^_TA_), the maximum frequency of the out-of-plane acoustic branch (*ω*^m^_ZA_), the maximum group velocity of the longitudinal acoustic branch (*V*^m^_LA_), the maximum group velocity of the transverse acoustic branch (*V*^m^_TA_), and the maximum group velocity of the out-of-plane acoustic branch (*V*^m^_ZA_) of the s/o-bismuth and β-bismuth monolayer

Materials	*ω* ^m^_LA_ (THz)	*ω* ^m^_TA_ (THz)	*ω* ^m^_ZA_ (THz)	*V* ^m^_LA_ (km s^−1^)	*V* ^m^_TA_ (km s^−1^)	*V* ^m^_ZA_ (km s^−1^)	Method
s/o-Bismuth	0.67	0.49	0.49	4.49	3.22	2.75	This work
β-Bismuth	1.16	0.98	0.77	19.26	11.30	6.86	Ref. 18

### 3.3 Electronic transport properties and figure of merit of s/o-bismuth monolayer

In order to investigate the figure of merit of the s/o-bismuth monolayer, the relaxation time of the carriers (electrons and holes) should be calculated beforehand. In the present study, the deformation potential theory (DP), which only includes the matrix elements of interactions between electrons and longitudinal acoustic phonons,^49,50^ was performed to calculate the relaxation time of the s/o-bismuth monolayer. It should be pointed out that the relaxation time of the s/o-bismuth monolayer would be reliable using the DP theory, which has been successfully applied in many 2D materials.^51,52^

According to the DP theory, the relaxation time (*τ*_β_) along a certain direction *β* (*β* = *x*, *y*) for the two-dimensional system can be derived by the following form:^26,53–55^6
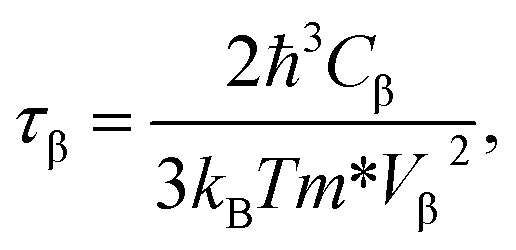
where m* is the effective masses of electrons or holes, *C*_β_ and *V*_β_ are elastic constant and deformation potential constant along a certain direction *β* (*β* = *x*, *y*), respectively. The values of m*, *C*_β_, and *V*_β_ are calculated as follows:7
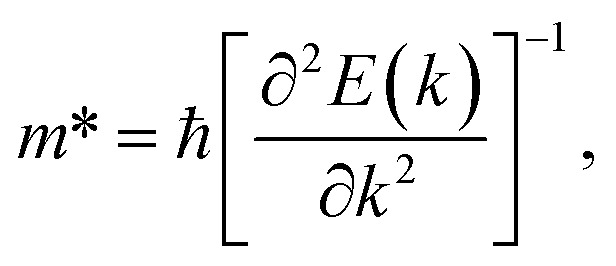
8
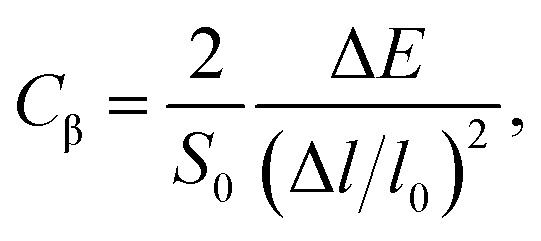
9
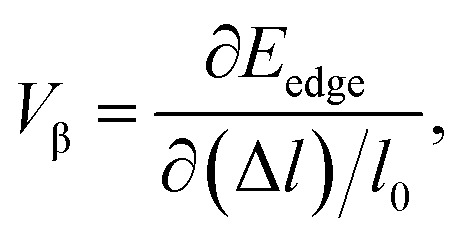
where *C*_β_ is the elastic constants of the s/o-bismuth monolayer; Δ*E* is the total energy change due to Δ*l*/*l*_0_, in which *l*_0_ is the equilibrium lattice spacing along the direction *β*, and Δ*l* = *l* − *l*_0_ is the change of the lattice spacing; *S*_0_ is the surface area of the s/o-bismuth monolayer; *E*_edge_ is the valence band maximum or conduction band minimum, and *V*_β_ is the deformation potential constant that represents the shift of band edge per unit strain.

After a series of calculations, the relaxation time of the s/o-bismuth monolayer was derived as a function of temperature. As a typical example, Table 4 shows the obtained effective masses m*, elastic constants *C*_β_, deformation potential constant *V*_β_, and relaxation time *τ*_β_ along a certain direction *β* (*β* = *x*, *y*) of the s/o-bismuth monolayer as well as the β-bismuth monolayer for the sake of comparison at 300 K. It should be pointed out that the *x* and *y* directions of the s/o-bismuth monolayer are shown in Fig. 1(a). One could discern clearly from Table 4 that the effective mass of the holes in the s/o-bismuth monolayer is bigger than the corresponding value in the β-bismuth monolayer. On the other hand, the effective mass of electrons of these two different monolayers is approaching each other. One could also discern from Table 4 that for the s/o-bismuth monolayer, there is a sharp decrease of elastic constants compared with the β-bismuth monolayer, and such a decrease in elastic constants would bring about weakened bond strength and subsequent low phonon velocity, which are consistent with the lower lattice thermal conductivity revealed in Section IIIB. Moreover, as shown in Table 4, for both electrons and holes, the present relaxation times along the *x* or *y* direction of the s/o-bismuth monolayer are lower than those of the corresponding β-bismuth monolayer, which suggests a weaker coupling of electrons and phonons in the s/o-bismuth monolayer and contributes to its better thermoelectric performance.^56^

**Table 4:** Elastic constant *C*_*i*_, DP constants *V*_*i*_, effective mass m*, and relaxation time *τ* (at 300 K) along the different orientation of the square/octagon (s/o) bismuth monolayer and the orthogonal supercell of the β-bismuth monolayer

Mater type		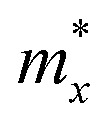 (*m*_0_)	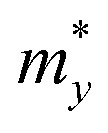 (*m*_0_)	*C*_*x*_ (N m^−1^)	*C*_*y*_ (N m^−1^)	*V*_*x*_ (eV)	*V*_*y*_ (eV)	*τ*_*x*_ (ps)	*τ*_*y*_ (ps)	Method
s/o-Bi	h	2.15	2.16	12.64	12.64	5.51	5.51	0.002	0.002	LDA + SOC
	e	0.11	0.11	12.64	12.64	2.88	2.88	0.11	0.11	LDA + SOC
β-Bi	h	0.23	0.23	23.86	23.96	1.61	9.14	0.52	0.09	LDA + SOC^18^
	e	0.14	0.14	23.86	23.96	3.08	3.49	0.44	0.39	LDA + SOC^18^

**Fig. 8:**
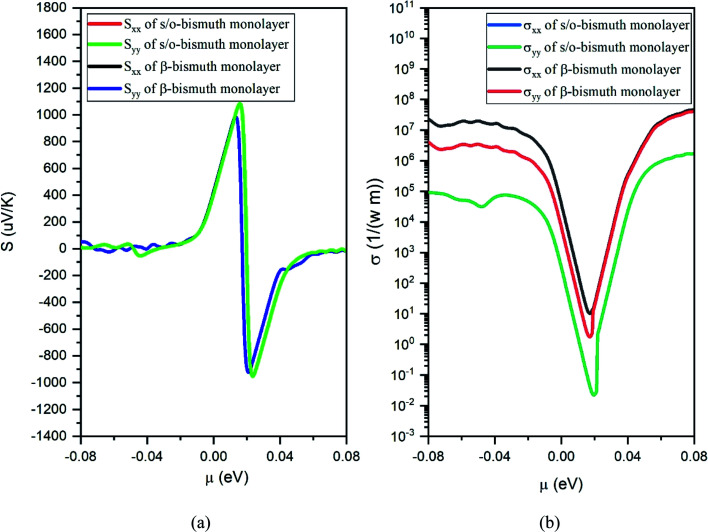
Seebeck coefficients (a) and the electronic conductivity (b) of the s/o-bismuth monolayer and β-bismuth monolayer structure^18^ as the function of chemical potential (*μ*), respectively.

**Fig. 9:**
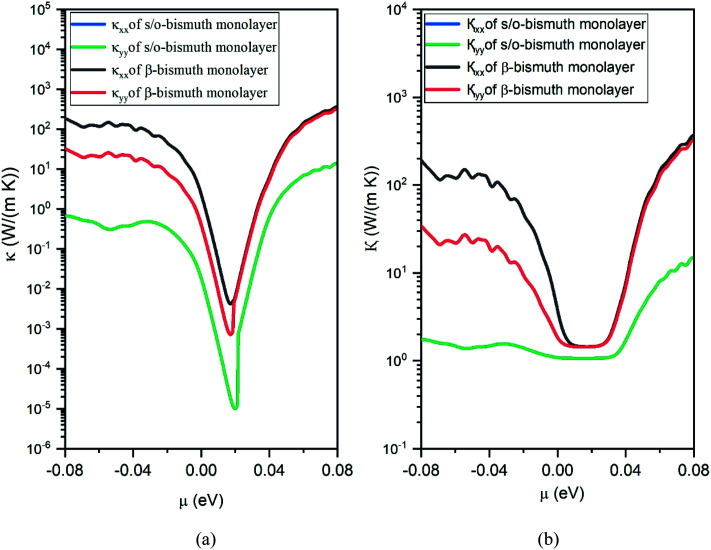
Electronic thermal conductivity (a) and Total thermal conductivity (b) of the s/o-bismuth monolayer and β-bismuth monolayer structure^18^ as the function of chemical potential (*μ*) at 300 K, respectively.

After obtaining the obtained relaxation time, the electrical conductivity (*σ*), electric thermal conductivity (*κ*_e_), and total thermal conductivity (*κ* = *κ*_e_ + *κ*_L_) of the s/o-bismuth monolayer are calculated as a function of chemical potential as well as the Seebeck coefficients (*S*). Thereby, the figure of merit of the s/o-bismuth monolayer can be calculated according to eqn (1). As typical examples, Fig. 8, 9, and 10 display the Seebeck coefficients, electrical conductivity (*σ*), electric thermal conductivity (*κ*_e_), total thermal conductivity (*κ*), and figure of merit (*ZT*) of the s/o-bismuth monolayer as the function of chemical potential (*μ*) as well as the β-bismuth monolayer in the literature^18^ for the sake of comparison.

**Fig. 10:**
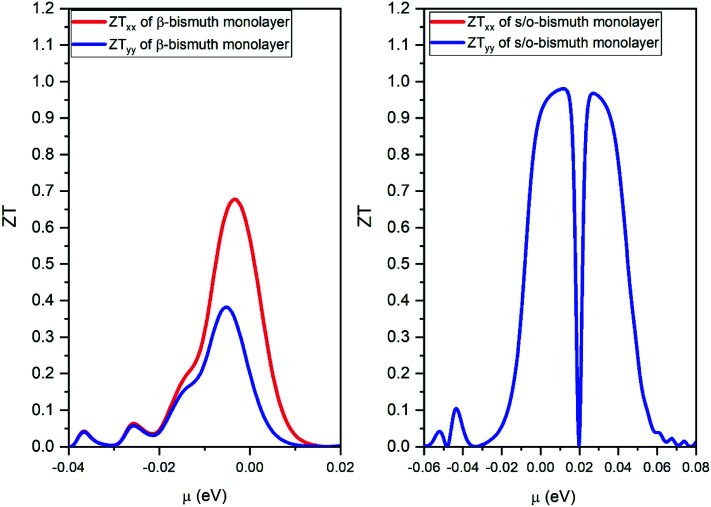
The figure of merit of p- and n-type of the s/o-bismuth monolayer along *x* and *y* orientation and the orthogonal supercell of the β-bismuth monolayer along *x* and *y* orientation^18^ as the function of chemical potential (*μ*) at the 300 K, respectively.

Several characteristics could be discerned from Fig. 8, 9, and 10. First of all, the absolute values of the maximum Seebeck coefficients of the s/o-bismuth monolayer are 1084, 1084, 952, and 952 μV K^−1^ for electrons and holes along the *x* and *y* directions, respectively, which are slightly larger than the corresponding values of 967, 967, 898, and 898 μV K^−1^ of the β-bismuth monolayer.^18^ The higher Seebeck coefficient of the s/o-bismuth monolayer for holes can be understood by the larger effective mass (2.16*m*_o_) and the presence of fewer minority carriers in the conduction band owing to the higher *E*_VBM_ (−0.11888 eV) than that of the β-bismuth monolayer, while the higher Seebeck coefficient of the s/o-bismuth monolayer for electrons may be due to the presence of fewer minority carrier due to the lower *E*_CBM_ (0.16452 eV) than that of the β-bismuth monolayer based on the above-calculated band structure and the effective mass, which matches well with the similar theoretical conclusion in the literature.^39^

Secondly, the electrical conductivity (*σ*) and electric thermal conductivity (*κ*_e_) of the s/o-bismuth monolayer are slightly lower than those of the β-bismuth monolayer, which indicates better electronic transport properties of the s/o-bismuth monolayer. In addition, the total thermal conductivity (*κ*) of the s/o-bismuth monolayer is lower than that of the β-bismuth monolayer, which may be probably attributed to the lower lattice and electronic thermal conductivity of s/o-bismuth monolayer as shown before in Fig. 9(a). The shape of the curve of electronic thermal conductivity in Fig. 9(a) is very similar to that of the electronic conductivity in Fig. 8(b), which is consistent with the Wiedemann–Franz law.^57^

Thirdly, the *ZT* values of the holes and electrons of the s/o-bismuth monolayer along the *x* or *y* direction are all much higher than the corresponding values of the β-bismuth monolayer, respectively. Interestingly, the maximum *ZT* value of the s/o-bismuth monolayer for holes (0.974) and electrons (0.973) is approaching each other, and both are higher than the corresponding maximum *ZT* value of β-bismuth monolayer (0.69).^18^ Additionally, this high figure of merit may have contributed to the governing factor such that s/o-bismuth monolayer has unusually lower lattice thermal conductivity and weaker coupling of electrons and phonons than those of the β-bismuth monolayer.

## 4. Conclusions

In summary, highly accurate first-principles calculation and Boltzmann transport theory have been used to reveal electronic structures, phonon band structures, and thermoelectric properties of the s/o-bismuth monolayer. Results show that the lattice thermal conductivity of the s/o-bismuth monolayer is lower than that of the β-bismuth monolayer owing to the lower phonon velocity. However, the Grüneisen parameter, the total phase space for three-phonon processes, and the phonon volumetric specific heat (*c*_ph_) are only negligible factors in the lattice thermal conductivity. In addition, the maximum value of the figure of merit of the s/o-bismuth monolayer is 0.974, which should be much larger than that of the β-bismuth monolayer. The derived results are in good agreement with other theoretical results in the literature, and could provide a deep understanding of the thermoelectric properties of the bismuth monolayer materials.

## Compliance with ethical standards

Funding: Project supported by the Natural Science Foundation of Hunan Province (Grant No. 2020JJ4236), Huxiang Youth talent project of Hunan Province (Grant no. 2018RS3099), Project supported by the Research Foundation of Education Bureau of Hunan Province (Grant No. 20A103), China.

## Conflicts of interest

The authors declare that they have no conflict of interest.

## Supplementary Material
